# An extensive bioinformatics study on the role of mitochondrial solute carrier family 25 in PC and its mechanism behind affecting immune infiltration and tumor energy metabolism

**DOI:** 10.1186/s12967-022-03756-2

**Published:** 2022-12-13

**Authors:** Qiang Zhang, Yubao Tang, Shuai Sun, Qiuyi Xie, Jie Yao, Xiaodong Wang, Jianjun Qian, Zhennan Li

**Affiliations:** 1 Medical College of Yangzhou University, Yangzhou, Jiangsu 225000 China; 2 Dalian Medical University, Dalian, 111600 Liaoning China; 3 Department of Hepatobiliary and Pancreatic Surgery, Northern Jiangsu People’s Hospital, Yangzhou, 225001 Jiangsu China

## Abstract

**Background:**

Several metabolic disorders and malignancies are directly related to abnormal mitochondrial solute carrier family 25 (SLC25A) members activity. However, its biological role in pancreatic cancer (PC) is not entirely understood.

**Methods:**

The lasso method was used to create a novel prognostic risk model for PC based on SLC25A members, and its roles in tumor immunology and energy metabolism were explored. Furthermore, co-expression networks were constructed for *SLC25A11, SLC25A29,* and *SLC25A44*. Single-cell RNA sequencing (ScRNA-seq) revealed the distribution of gene expression in PC. Tumor immune infiltration was examined with the TIMER database. Lastly, drug sensitivity was investigated, and co-transcriptional factors were predicted.

**Results:**

In the present study, a novel prognostic risk model was established and validated for PC based on SLC25A members. The high-risk group had a lower activation of oxidative phosphorylation and a more abundant immune infiltration phenotype than the low-risk group. According to co-expression network studies, *SLC25A11, SLC25A29,* and *SLC25A44* were involved in the energy metabolism of PC and prevented tumor growth, invasion, and metastasis. ScRNA-seq research also pointed to their contribution to the tumor microenvironment. Moreover, the recruitment of numerous immune cells was positively correlated with *SLC25A11* and *SLC25A44* but negatively correlated with *SLC25A29*. Additionally, the sensitivity to 20 Food and Drug Administration-approved antineoplastic medicines was strongly linked to the aforementioned genes, where cisplatin sensitivity increased with the up-regulation of *SLC25A29*. Finally, the *Scleraxis BHLH Transcription Factor (SCX)* and other proteins were hypothesized to co-regulate the mRNA transcription of the genes.

**Conclusion:**

SLC25A members are crucial for tumor immune and energy metabolism in PC, and *SLC25A11*, *SLC25A29,* and *SLC25A44* can be used as favorable prognostic markers. The use of these markers will provide new directions to unravel their action mechanisms in PC.

**Supplementary Information:**

The online version contains supplementary material available at 10.1186/s12967-022-03756-2.

## Introduction

Pancreatic cancer (PC) is one of the most malignant cancers. According to GLOBOCAN, nearly 466,000 people die from PC annually, which is almost equal to the number of diagnosed cases (496,000). The 5-year survival rate is < 8%, which makes PC the seventh most important cause of cancer-related death worldwide [1]. The deep anatomical position of the pancreas and the lack of typical symptoms and effective diagnostic methods in the early stage cause nearly 80%–90% of the patients to miss the optimal surgical treatment period [2, 3]. In addition, the benefits derived by patients with PC from comprehensive treatment strategies, such as systemic chemotherapy and targeted therapy, are extremely poor [4]. Therefore, the molecular mechanism of PC needs to be further elucidated.

Mitochondria are biological organelles with self-replicable and transcribable DNA, and mitochondrial DNA (mtDNA) lesions are an important cause of disease [5]. Furthermore, transport of metabolites through mitochondrial membrane is a critical phase in the process of mitochondria partaking in cellular oxidation and energy output, and this step is heavily regulated by inner membrane [6]. The solute carrier family is chiefly located on the membranes of cells and organelles and is the second largest group of membrane transporters after G protein-coupled receptors [7]. SLC25A members, the largest solute carrier group of the solute carrier family, contains a highly specific sequence of six counter-clockwise-aligned transmembrane α-helices and three repetitive short α-helices [8]. This sequence links several metabolic processes (e.g., oxidative phosphorylation and citric acid cycle) among different cellular compartments (cytoplasmic matrix and mitochondrial matrix) by mediating solute translocation across the membrane [9]. Recent studies have shown that SLC25A members are involved in multiple processes of tumorigenesis, including metabolic reprogramming, mitochondrial apoptosis, maintenance of cellular redox homeostasis, the transformation of metastatic phenotypes, and promotion of cancer stem cell stemness and chemoresistance [10–18]. However, the biological role of SLC25A members and its mechanism of action in PC have not been entirely elucidated.

In the present study, differences in the expression and function of SLC25A members in PC and its correlation with tumor immune infiltration were analyzed. Furthermore, a prognostic risk model was constructed using public databases. In addition, the functions of the identified SLC25A members (*SLC25A11*, *SLC25A29,* and *SLC25A44)* in PC were explored, and transcription factor (TF) networks were created based on them. Considering the heterogeneity of the tumor cells, the role of SLC25A members in the tumor microenvironment (TME) was also analyzed using single-cell RNA-seq (scRNA) data.

## Results

### The role of the SLC25A members in PC

**Fig. 1:**
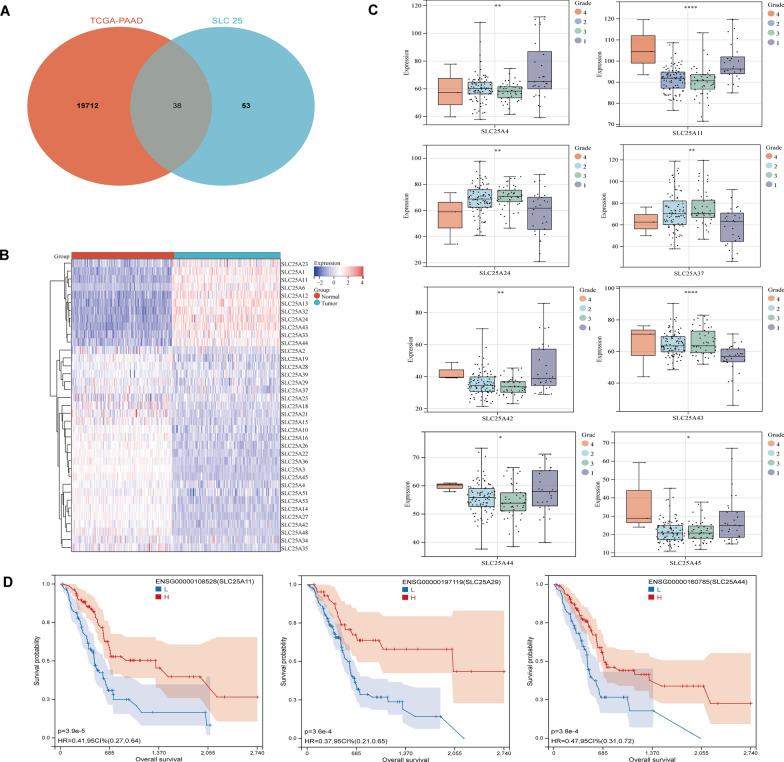
Preliminary investigation of mitochondrial solute carrier family 25 (SLC25A) members in PC. **A** Venn diagram depicting the interaction between SLC25A members and differentially expressed genes (DEGs) from the TCGA-PAAD cohort (Normal vs. Tumor). **B** In comparison to normal pancreatic tissues, 11 SLC25A members were downregulated and 27 SLC25A members were upregulated in the PC tumor tissues (**C**). The differential expression levels of 38 SLC25A members between different neoplasm histologic grades (1: well differentiation, 2: moderate differentiation, 3: poor differentiation, 4: undifferentiation), (^**−**^*P* > *0.05,* *P < *0.05,* **P < *0.01,* ***P < *0.001,* ****P < *0.0001*). **D** High expression of SLC25A11, SLC25A29, and SLC25A44 was associated with longer patient survival in PC, according to Kaplan–Meier analysis

The TCGA-PAAD cohort was collected (177 tumor tissues and 4 normal tissues) from the TCGA database, and 167 normal pancreatic tissue samples were pooled from the GTEx database. By analyzing the differential expression of genes between PC tumor tissue and normal tissue, a total of 19,712 differentially expressed genes (DEGs) were identified (up-regulated: 10,973, down-regulated: 8739). Finally, 38 DEGs of SLC25A members were screened using the Venn diagram (Fig. 1A). Compared with normal pancreatic tissues, 11 genes were down-regulated and 26 genes were up-regulated in PC tumor tissues, as demonstrated using heatmap (Fig. 1B, Additional file 1: Table. S1).

To investigate the correlation between SLC25A members and the clinical features of PC, the differences in the expression levels of DEGs of SLC25A members among various tumor pathological grades were examined in the TCGA-PAAD cohort. As shown, of the various tumor pathological grades, the expression levels of *SLC25A4* (P < 0.01), *SLC25A11* (P < 0.0001), *SLC25A24* (P < 0.01), *SLC25A37* (P < 0.01), *SLC25A42* (P < 0.01), *SLC25A43* (P < 0.0001), *SLC25A44* (P < 0.05) and *SLC25A45* (P < 0.05) were significantly different (Fig. 1C). Furthermore, the prognostic value of *SLC25* DEGs in PC was investigated. The results showed that patients with PC who showed high expressions of *SLC25A4* (P = 0.01), *SLC25A6* (P = 3.9e−3), *SLC25A11* (P = 3.9e−5), *SLC25A14* (P = 1.8e−3), *SLC25A27* (P = 1.5e−3), *SLC25A29* (P = 3.6e−4), *SLC25A34* (P = 8.9e−4), *SLC25A42* (P = 9.7e−3), *SLC25A44* (P = 3.8e−4), *SLC25A45* (P = 4.1e−3) and *SLC25A53* (P = 6.3e−5) had better prognosis. In contrast, those with low expressions of *SLC25A24* (P = 9.3e−3), *SLC25A37* (P = 0.02) and *SLC25A43* (P = 3.63e−3) had longer survival (Fig. 1D, Additional file 2: Fig. S1C). Finally, we reveal that the DEGs of SLC25A members significantly correlate with each other (negative: blue; positive: red). Similar to that, there is a considerable protein-level interaction between them (Additional file 2: Fig. S1A, B).

### The performance of the new classification and prognostic risk model based on SLC25A members in PC

**Fig. 2:**
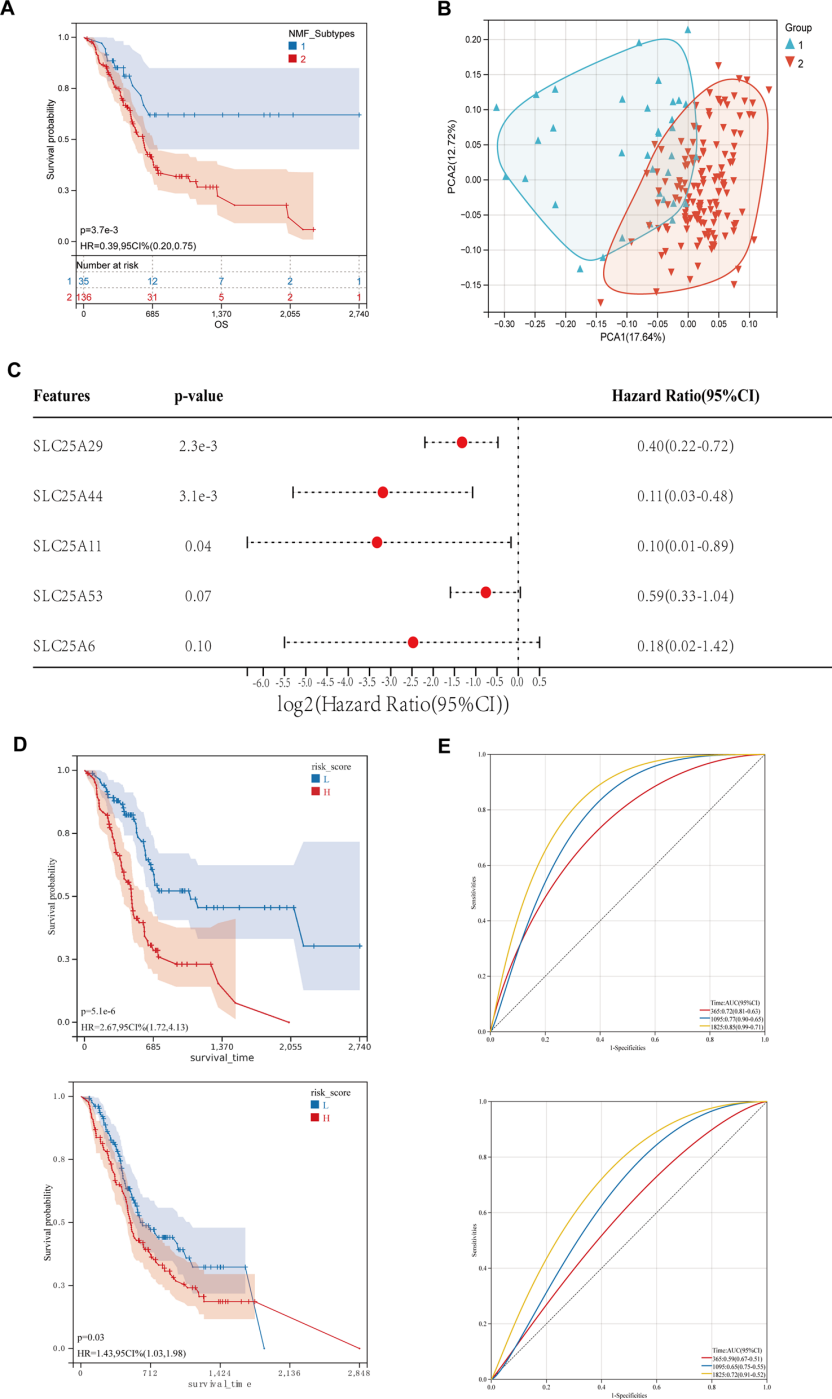
Identification of a new classification of mitochondrial solute carrier family 25 (SLC25A) members and the construction of a prognostic risk model in PC. **A** Survival analysis revealed that the new classification had a good prognostic value in the TCGA-PAAD cohort. **B** The principal component analysis detected a partial overlap between clusters 1 and 2, and it did not distinguish well between PC patients based on the mRNA expression levels. **C** A new 5-signature prognostic model for PC based on DEGs of SLC25A members was developed using Lasso and Cox multifactor regression analysis. **D** In the TCGA-PAAD cohort, the new prognostic model demonstrated a significant prognostic value, which was verified in the ICGC-PAAD cohort. **E** The area under the curve (AUC) for 1-, 3-, and 5-year OS in the TCGA cohort was 0.72, 0.77, and 0.85, respectively, according to the Receiver Operating Characteristic (ROC) findings. Next, we assessed the model's predictive abilities in the ICGC-PAAD cohort, and the findings revealed improved predictive efficacy

To gain a more comprehensive understanding of the role of SLC25A members in PC, the DEGs were re-clustered based on SLC25A members to identify two novel subtypes while ensuring optimal stability (Fig. S2A, B). Survival analysis revealed that survival was significantly longer in the TCGA-PAAD cohort in Cluster 1 (blue) than in Cluster 2 (red) (Fig. 2A). However, principal component analysis (PCA) indicated a partial overlap between Clusters 1 and 2, and patients with PC could not be distinguished well based on mRNA expression levels (Fig. 2B).

Therefore, a prognostic risk model was constructed to evaluate the role of SLC25A members in PC more comprehensively. The expression data of 14 prognosis-related DEGs of SLC25A members in the TCGA-PAAD cohort were integrated (Fig. 1D, Additional file 2: Fig. S1C), and regression analysis was then performed using a lasso. Subsequently, tenfold crossover validation was done, and finally, 10 genes were screened out (Additional file 1: Table S2). These genes were then subjected to Cox multivariate regression analysis of subsequent stepwise regression, and the optimal model with five genes was obtained (Fig. 2C, Additional file 3: Fig. S2C). The samples were categorized into high- and low-risk groups according to the median of the model’s risk score. Survival analysis alluded that the patients in the high-risk group had shorter survival (P = 5.1e−6) (Fig. 2D). According to the receiver operating characteristic (ROC) curve results, the areas under the curve (AUC) for the 1-, 3- and 5-year overall survival (OS) were 0.72, 0.77 and 0.85, respectively, in the TCGA cohort. This finding suggests that the model has a good predictive value for the survival time of the TCGA cohort (Fig. 2E). The predictive ability of the model for the ICGC-PAAD cohort was validated, and the results showed good predictive performance.

### Significant differences in tumor immune infiltration phenotype and energy metabolic pathways levels between different risk groups

**Fig. 3:**
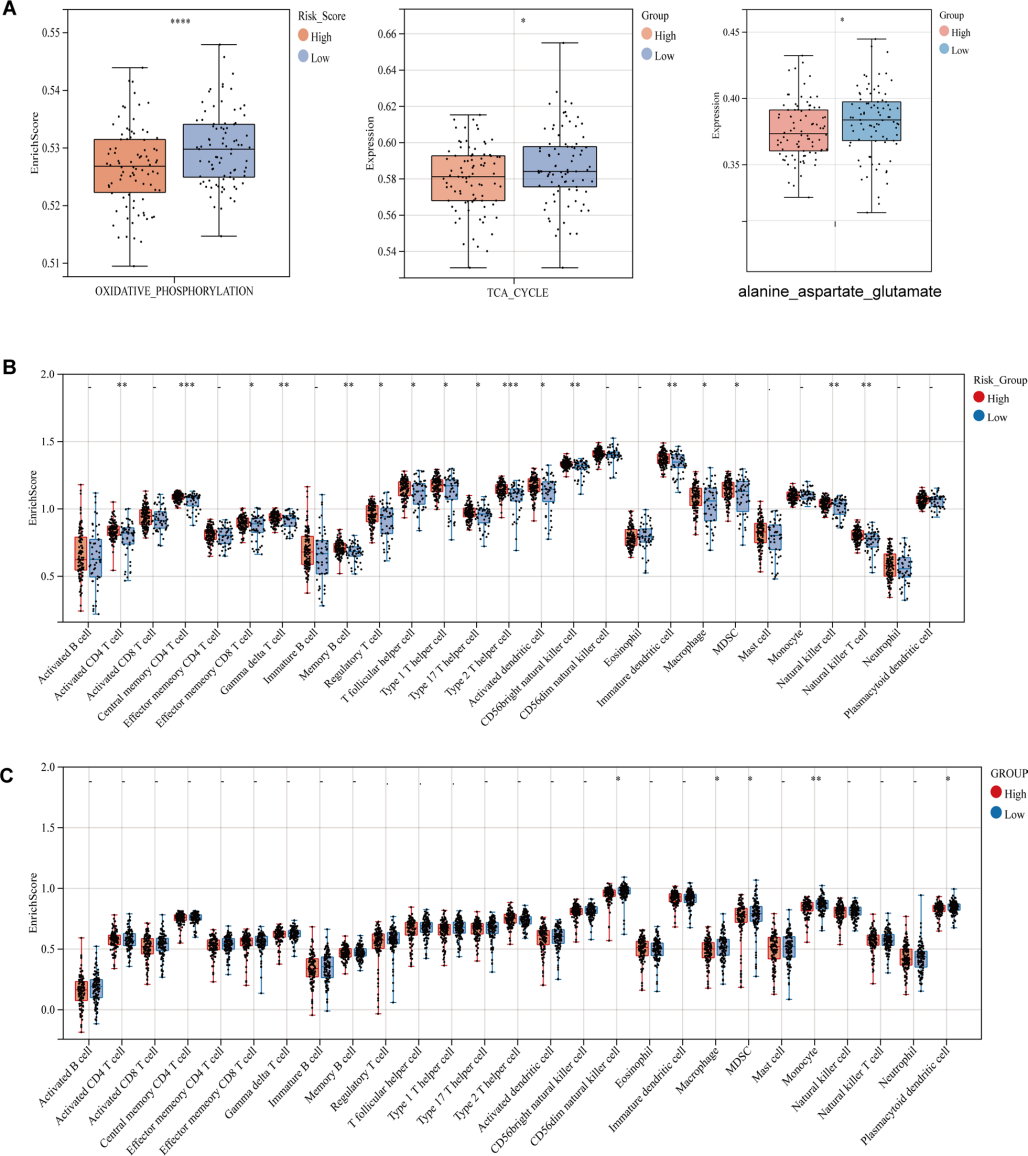
High-risk group associated with tumor immune-infiltration phenotype and a low level of oxidative phosphorylation. **A** The study of single-sample gene set enrichment (ssGSEA) assesses the activity of oxidative phosphorylation, TCA cycle and alanine_aspartate_glutamate metabolic pathway in each PC sample and analyses the differences in the levels of this pathway between high- and low-risk groups, (^**−**^*P* > *0.05,* *P < *0.05,* **P < *0.01,* ***P < *0.001,* ****P < *0.0001*). **B** The differences in the level of infiltration of 29 immune cell species between different risk groups were examined using ssGSEA in the TCGA-PAAD cohort. **C** The differences in the level of infiltration of 29 immune cell species between different risk groups were examined using ssGSEA in the ICGC-PAAD cohort

To further elucidate the role of the SLC25A-based prognostic risk model in PC, the infiltration of immune-infiltrating phenotypes was explored in the high- and low-risk groups. The results showed that the immune-infiltrating cells were more abundantly characterized in tumors in the high-risk group than in the low-risk group (Fig. 3B, 3C). It is well known that the main energy metabolism of PC cells is oxygen-consuming glycolysis. Oxidative phosphorylation (OxPhos) and tricarboxylic acid (TCA) cycle pathway scores were greater in the low-risk group than in the high-risk group (Fig. 3A). Amino acid metabolism was further examined, which revealed that alanine_aspartate_glutamate metabolic pathway scores were significantly higher in the low-risk group than in the high-risk group. This finding suggests that SLC25A members may be involved in the metabolic remodeling of PC tumor cells.

### Risk score of the SLC25A-based risk model is an independent risk factor for predicting PC prognosis

**Fig. 4:**
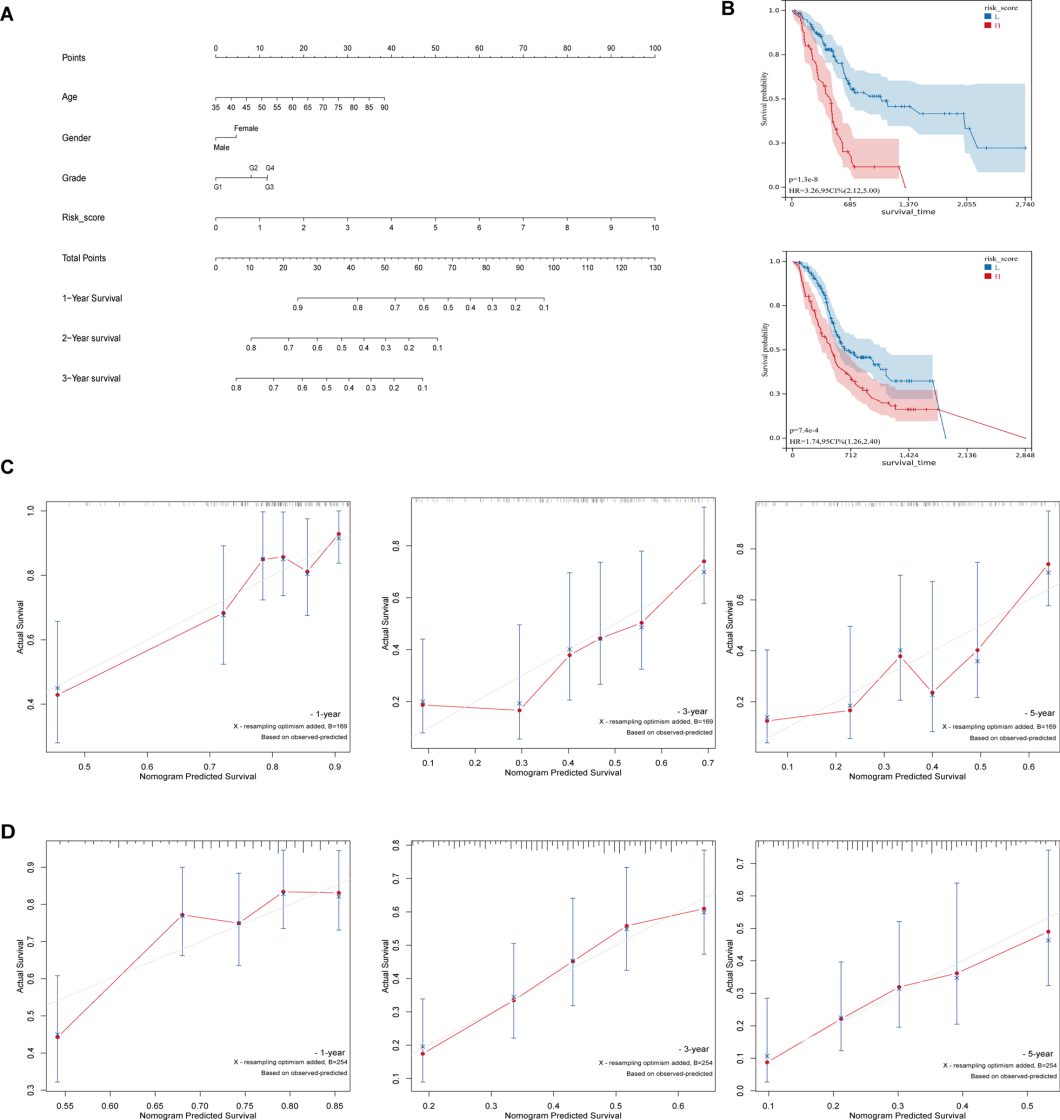
A risk score based on SLC 25 prognostic model as an independent risk factor for PC. **A** A nomogram with risk ratings and prognostic indicators for TCGA-PAAD patients. **B** Patients with high-risk scores exhibited relatively shorter OS time in both the TCGA-PAAD cohort and the ICGC-PAAD cohort, according to Kaplan–Meier analyses. **C**, **D** The calibration curves of the nomogram demonstrated the highest concordance between the observed clinical outcomes of PC patients in the TCGA and ICGC datasets and the anticipated prognostic outcomes after 1, 2, and 3 years

After integration of age, gender, tumor grade, and risk score in the TCGA-PAAD and PAAD-CA cohorts, multifactorial Cox regression analysis showed that risk score (P < 0.001) was an independent factor for predicting PC prognosis (Fig. 4B). Calculation of the C-index showed that the model had good discrimination in the TCGA-PAAD [0.678 (95% confidence interval (CI) 0,614–0.747)] and ICGC-PAAD [0.647 (95% CI 0.600–0.694)] cohorts. Moreover, nomogram containing the risk scores were constructed using the clinical characteristics to obtain the predictive values of 1-, 2- and 3-year prognosis of patients with TCGA-PAAD (Fig. 4A). To facilitate scholarly applications, a web version of the dynamic nomogram (minnietree.shinyapps.io/DynNomapp) was deployed. Finally, the calibration curves showed good agreement between the training and validation sets in the predicted prognostic outcomes as well as the actual observed clinical outcomes of patients with PC at 1, 2, and 3 years (Fig. 4C, D).

### Co-expression network analysis of *SLC25A11, SLC25A29,* and *SLC25A44*

**Fig. 5:**
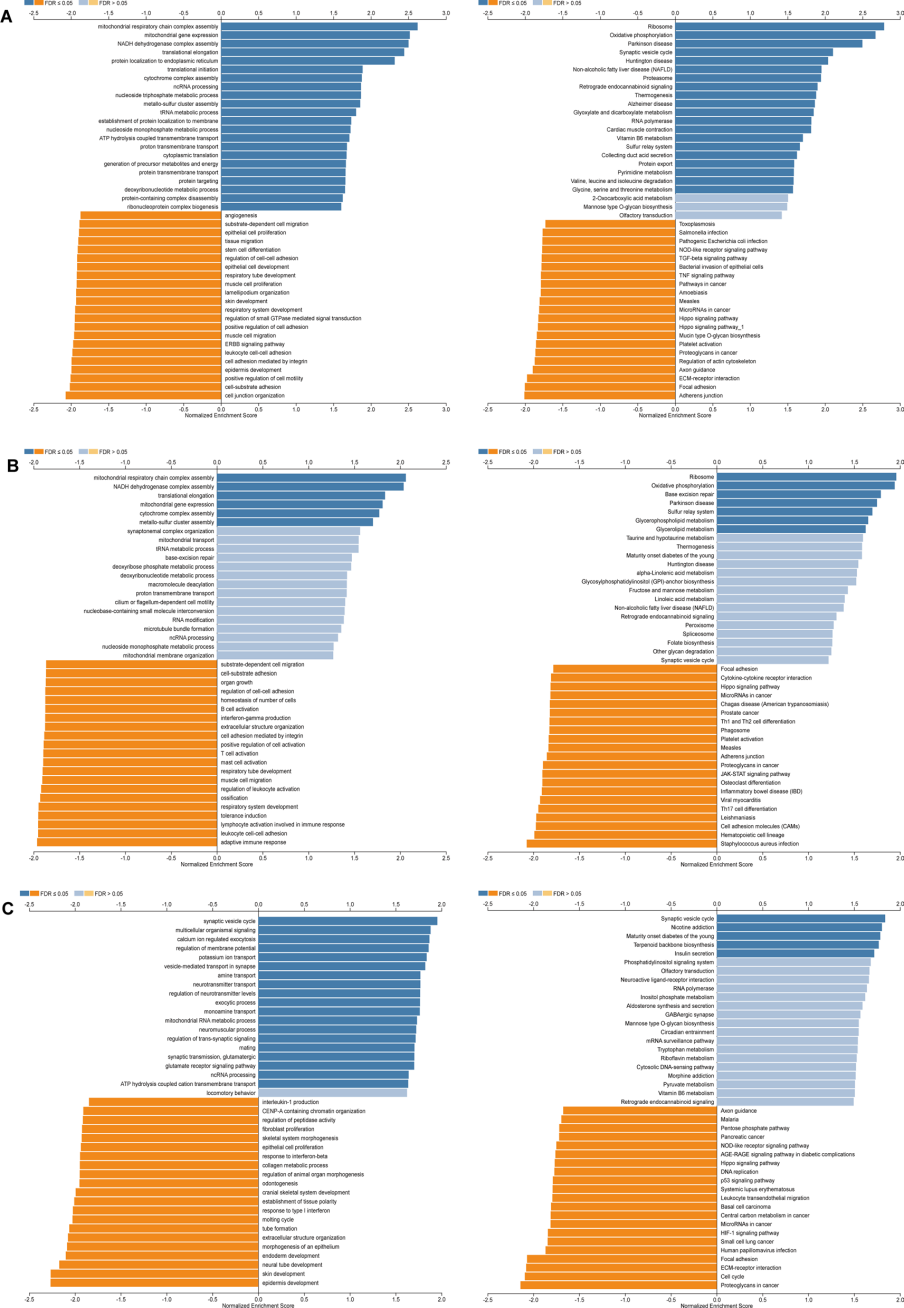
Co-expression genes set variation analysis (GSVA) of SLC25A11, SLC25A29, and SLC25A44 (Gene Ontology (GO) and signaling pathways in Kyoto Encyclopedia of Genes and Genomes (KEGG)). **A**–**C** The GO_BP and KEGG pathways (GSEA) of SLC25A11, SLC25A29, and SLC25A44 co-expressed genes in PC were investigated

*SLC25A11*, *SLC25A29,* and *SLC25A44* were screened for differential expression in PC, with significant prognostic value, in previous univariate and multifactorial Cox regression analyses. Subsequently, separate in-depth analyses of the genes were conducted to explore their functions in PC. Functional module analysis based on LinkedOmics indicated that the transcriptome expression level of *SLC25A11* in PC was positively correlated with the expression of 5057 genes but negatively correlated with the expression of 5069 genes (FDR < 0.05); the expression level of *SLC25A2*9 was positively correlated with the expression of 4960 genes and negatively correlated with the expression of 5937 genes (FDR < 0.05); the expression level of *SLC25A44* was positively correlated with the expression of 2106 genes and negatively correlated with the expression of 1835 genes (FDR < 0.05) (Additional file 4: Fig. S3A). The top 50 differentially co-expressed genes are portrayed in the heatmap (Additional file 4: Fig. S3B, C, D). Gene Ontology (GO) terminology annotation of gene enrichment analysis illustrated that *SLC25A11* and its positively correlated genes were mainly involved in the processes of mitochondrial respiratory chain complex assembly, mitochondrial gene expression, translational elongation, and protein localization to the endoplasmic reticulum. Conversely, the negatively correlated genes were involved in the processes of cell junction organization, cell-substrate adhesion, positive regulation of cell motility, and epidermis development. In the *SLC25A29* co-expression network, the positively correlated biological processes were essentially the same as those in *SLL25A11*, except for adaptive immune response, but it was negatively associated with multiple immune cell activation processes. Finally, *SLC25A44* and its positively correlated genes were mainly involved in the synaptic vesicle cycle, multicellular organismal signaling, calcium-ion-regulated exocytosis, and regulation of membrane potential. Furthermore, the Kyoto Encyclopedia of Genes and Genomes (KEGG) pathway analysis showed that the co-expressed positively correlated genes *SLC25A11* and *SLC25A29* were predominantly enriched in the ribosome and OxPhos pathways. *SLC25A44* and its positively correlated genes, on the contrary, were enriched in pathways related to the synaptic vesicle cycle, nicotine addiction, maturity-onset diabetes of the young, and synaptic vesicle recycling. The negatively correlated genes *SLC25A11*, *SLC25A29,* and *SLC25A44* were enriched in adherens junction, focal adhesion, and ECM-receptor interaction. Furthermore, *SLC25A29* expression was negatively associated with various pathways, such as Th17, Th1, and Th2 cell differentiation. Additionally, *SLC25A44* and its negatively correlated genes were involved in PC, cell cycle, HIF-1, P53, and Hippo pathways (Fig. 5A–C). The above findings imply that *SLC25A11*, *SLC25A29,* and *SLC25A44* are likely to exert a significant impact on energy metabolism, tumor immunity, proliferation, invasion, and metastasis of PC cells.

### *SLC25A11*,* SLC25A29,* and *SLC25A44* are correlated with the infiltration of various immune cells

**Fig. 6:**
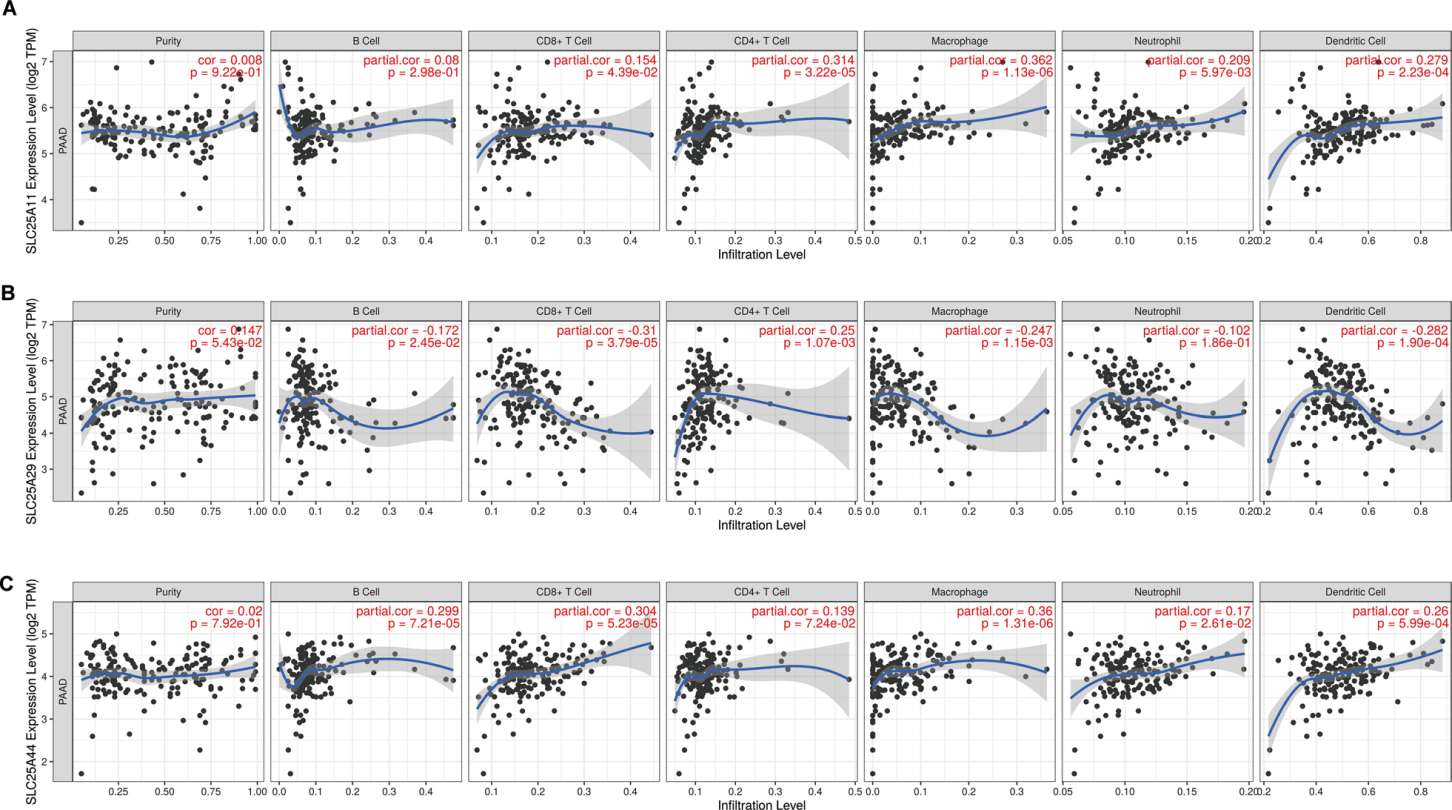
The abundance of tumor immune-cell infiltration correlates with the SLC25A11, SLC25A29, and SLC25A44 expression levels in PC. **A**–**C**. Timer immuno-infiltration analysis revealed that SLC25A11, SLC25A29, and SLC25A44 were significantly correlated with tumor immune cell recruitment

The correlation between *SLC25A11*, *SLC25A29,* and *SLC25A44* mRNA expression levels and tumor immune invasion was further explored in PC. *SLC25A11* demonstrated significant positive correlation (Fig. 6a) with CD8 + T cells (Partial. Cor = 0.154, P = 4.39E − 02), CD4 + T cells (Partial. Cor = 0.314, P = 3.22E − 05), macrophages (Partial. Cor = 0.362, P = 1.13E − 06), neutrophils (Partial. P = 5.97E − 03) and dendritic (DC) cells (Partial. Cor = 0.279, P = 2.23E − 04). *SLC25A29* presented significant negative correlation with B cells (Partial. Cor =  − 0.172, P = 2.45E − 02), CD8 + T cells (Partial. Cor = -0.31, P = 3.79E − 05), macrophages (Partial. Cor = -0.247, P = 1.15E − 03) and DC cells (Partial. Cor = − 0.282, P = 1.90E − 04). Moreover, a significant positive correlation was observed with the abundance of CD4 + T cells (Partial. Cor = 0.25, P = 1.07E − 03) (Fig. 6b). *SLC25A44* was found to be significantly positively correlated (Fig. 6c) with B cells (Partial. Cor = 0.299, P = 7.21E − 05), CD8 + T cells (Partial. Cor = 0.304, P = 5.23E − 05), macrophages (Partial. Cor = 0.36, P = 1.31E − 06), neutrophils (Partial. Cor = 0.17, P = 2.61e − 02) and DC cells (Partial. Cor = 0.26, P = 5.99E − 04). The above results suggest that *SLC25A11* and *SLC25A44* are involved in antitumor immunity, whereas *SLC25A29* is involved in pro-tumor immunity.

### The expressions of *SLC25A11*, *SLC25A29,* and *SLC25A44* in several PC cells

**Fig. 7:**
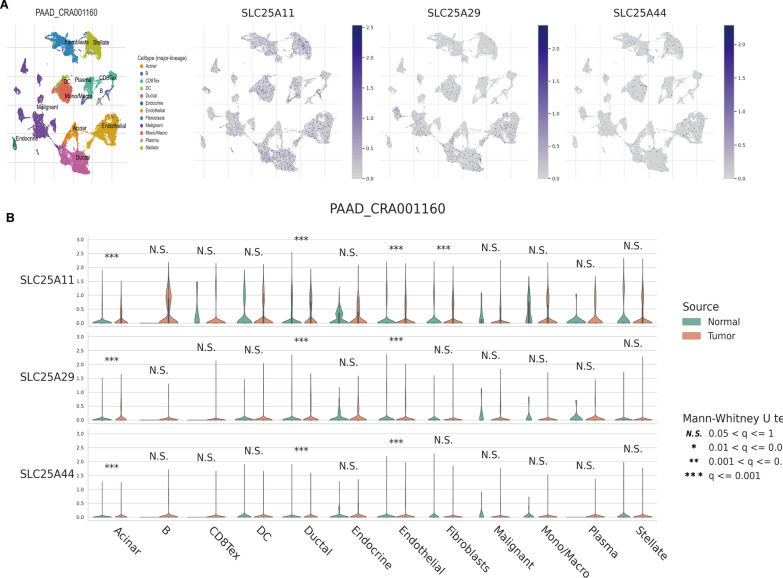
According to publicly available single-cell sequencing data, SLC25A11, SLC25A29, and SLC25A44 were all involved in the establishment of the PC tumor microenvironment***.*** **A** The UMAP plots revealed the relationship between the expression levels of different PC cell clusters and SLC25A11, SLC25A29, and SLC25A44, respectively. **B** Comparison of single-cell SLC25A11, SLC25A29, and SLC25A44 expression profiles between pancreatic normal and malignant tissues

To determine the potential mechanisms by which *SLC25A11*, *SLC25A29* and *SLC25A44* affect the TME in PC, their expression levels were analyzed in the TME of different cell types using a common scRNA dataset. *SLC25A11* and *SLC25A29* were noted to be expressed in stromal cells, malignant cells, and immune cells. *SLC25A44* was chiefly expressed in stromal cells (Fig. 7a). Interestingly, *SLC25A11*, *SLC25A29,* and *SLC25A44* were significantly overexpressed in ductal, acinar and endocrine cells in tumor tissues compared with normal tissues (P < 0.001) (Fig. 7b). These findings allude that *SLC25A11*, *SLC25A29,* and *SLC25A44* are closely related to the formation of the TME in PC.

### Polygenic TF networks and drug sensitivity analysis

**Fig. 8:**
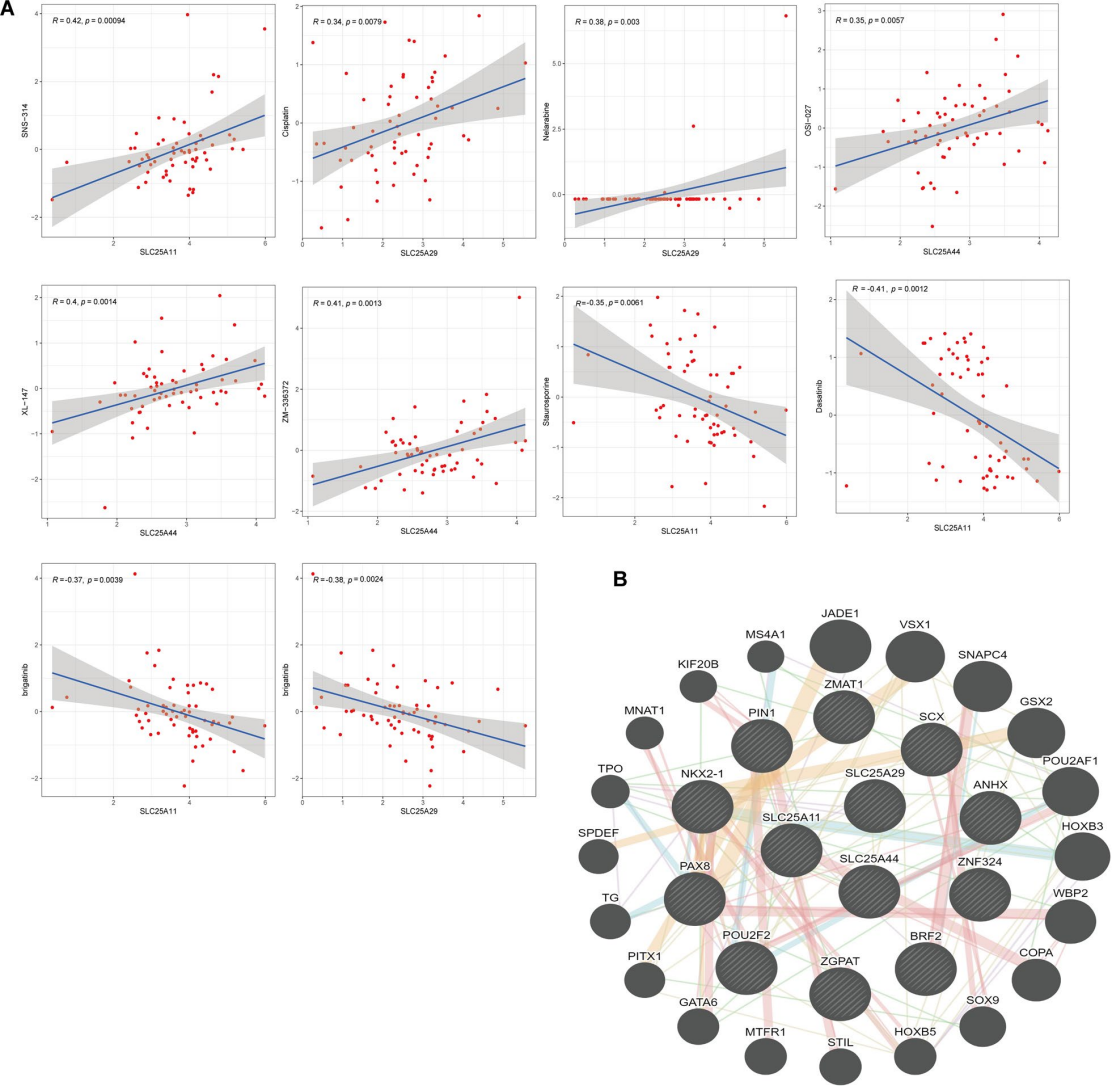
Multi-gene transcriptional regulator networks and drug sensitivity analysis. **A** Correlation of SLC25A11, SLC25A29, and SLC25A44 with the respective drug responses. **B** We examined the protein interaction network between the transcription factors and their target genes using GeneMANIA

To further understand the regulatory mechanisms of *SLC25A11*, *SLC25A29,* and *SLC25A44*, three common TFs were predicted using the CHEA3 database, namely, Top10 TFs of Mean Rank (*SCX, NK2 Homeobox 1* (*Nkx2-1*) et al.), which are most likely to be involved in the TFs of the above mRNA (Additional file 1: Table. S3). The protein interaction network between TFs and the target genes was analyzed using the GeneMANIA website (Fig. 8b).

Finally, the sensitivity of *SLC25A11, SLC25A29,* and *SLC25A44* to antitumor drugs was evaluated. The associations between *SLC25A11, SLC25A29,* and *SLC25A44* expression levels and 20 current Food and Drug Administration-approved anticancer drugs were assessed. The results showed (Fig. 8a) that the sensitivity to SNS-314 increased with the up-regulation of *SLC25A11*. The sensitivity to cisplatin and nellabine increased with the up-regulation of *SLC25A29*. Similarly, the sensitivity to XL-147, ZM-336372, and OSI-027 increased with the up-regulation of *SLC25A44*. Furthermore, the resistance to stellaria and dasatinib increased with the up-regulation of *SLC25A11*. The resistance to the drug trametinib increased with the up-regulation of *SLC25A29*, and the resistance to the drug bugatinib increased with the up-regulation of *SLC25A11* and *SLC25A29*.

## Discussion

In this study, based on the differential expression of SLC25A members in PC, its key role and potential mechanism in carcinogenesis and PC progression were analyzed using multi-omics techniques. A total of 38 SLC25A genes were found to be differentially expressed in PC, and some of them showed prognostic value in the disease. Subsequently, a prognostic risk model was constructed, which showed good predictive ability in both the training set and the validation cohort. Finally, the roles of *SC25A11, SLC25A29,* and *SLC25A44* in PC were explored, and all three genes were found to be up-regulated in PC tissues. Furthermore, their co-expressed genes were observed to be involved in the metabolic pathway of tumor tissues. The results of the immune infiltration analysis showed the presence of a significant correlation between the three factors and the abundance of various immune cell infiltrations. It is noteworthy that *SC25A11, SLC25A29,* and *SLC25A44* were observed to mainly exist in the malignant PC cells, stromal cells, and immune cells in the public scRNA database and were closely related to the formation of TME. Moreover, SCX and NKX2-1 were speculated to be the most likely related TFs among the three. Finally, the sensitivity to cisplatin and SNS-134 was found to increase with the up-regulation of *SC25A11, SLC25A29,* and *SLC25A44*.

SLC25A members are crucial in controlling how normally occurring pancreatic cells operate physiologically. In addition to producing ATP, mitochondria in pancreatic cells also produce metabolic substrates for glucose stimulated insulin secretion (GSIS) [19]. In this process, the phase by which pyruvate carboxylase transforms pyruvate into oxalacetic acid to promote the generation of crucial intermediates for TCA cycle function and insulin secretion is the limiting step [20]. Mitochondria citrate carrier (*SLC25A1*, CIC) contributes significantly to the above phases in two different mechanisms [21]. On the one hand, it makes citrate transport to the cytoplasm, and then through the cascade reaction to produce malonyl coenzyme A, to inhibit carnitine acyltransferase 1, resulting in an accumulation of acyl coenzyme A in the cytoplasm, thereby stimulating pancreatic GSIS. Isocitrate dehydrogenase activation, on the other hand, can encourage isocitrate breakdown to create -ketoglutaric acid and nicotinamide adenine dinucleotide phosphate (NADPH), which further stimulate pancreatic GSIS. Additionally, itaconate, which is synthesized from cytoplasmic citrate, has the potential to suppress the production of inflammatory mediators, controlling the metabolic pathway in inflammation, according to recent findings [15]. Therefore, we hope to reveal whether SLC25A members also play a potentially important role in pancreatic carcinogenesis and tumor immunity to some extent.

In this study, we hoped to clearly distinguish patients with PC via hierarchical clustering of non-negative matrix factorization (NMF). However, contrary to our expectations, although the new classification showed good prognostic significance in the TCGA-PAAD cohort, PCA revealed that it was not able to differentiate patients with PC based on mRNA expression levels. Therefore, a prognostic risk model for SLC25A members was established based on Cox multivariate analysis and lasso regression analysis. Validation was further performed on the PAAD-CA queue, and a rosette was constructed to calibrate the curve recognition model distinction. The rate of glycolysis has been reported to be higher in activated T cells as well as in differentiated Th1, Th2, and Th17 cells than in OxPhos [22]. In this study, a higher infiltration abundance of activated T-cells, Th1, Th2, and Th17 immune cells was observed in the high-risk group and, interestingly, lower OxPhos and TCA cycle activity were seen. This observation suggests that the associated immune cells influence the metabolic pattern of tumors in the high-risk group. Recent studies have shown that glutamine-derived aspartate is translocated to the cytoplasm via the oncogenic activation pathway of *KRAS* in pancreatic ductal adenocarcinoma cells. Subsequently, aspartate is converted to pyruvate and reduced NADPH via a sequential reaction, thus maintaining redox homeostasis in cancer cells [17]. Notably, alanine_aspartate_glutamate metabolism was found to be more active in the low-risk group. This result implies that tumors in the low-risk group are more involved in the maintenance of redox homeostasis than those in the high-risk group, but further validation is needed.

Since Warburg first proposed the hypothesis that cancer cells produce lactate from glucose because of mitochondrial destruction in 1956, ATP in cancer cells mainly comes from glycolysis, cancer metabolism research has entered a new era [23]. However, cancer energy metabolism is not as simple as assumed by Warburg. Studies have reported that inhibition of glycolytic ATP production by knocking down pyruvate kinase (PKM2) does not prevent tumorigenesis. This finding shows that mitochondrial OxPhos is still the main ATP supply pathway in various cancer cells despite the increase in glycolysis rate [24, 25]. Based on this observation, OxPhos has been proposed as a therapeutic target for cancers [26]. It is well known that in OxPhos, the functioning of proton pump and the production of ATP requires electron transport [27]. Kang Jinhua showed that cytoplasmic Nicotinamide Adenine Dinucleotide (NADH) is the main source of electrons in cancer cells, in which electrons are transferred from cytoplasmic NADH to mitochondrial NAD transport via the malate–aspartic acid shuttle, and finally, enter OxPhos [28].

*SLC25A11* is mitochondrial malate–α-ketoglutarate carrier protein, which is one of the two reverse transporters of the malate–aspartic acid shuttle. Our study found that *SLC25A11* and its positively related genes are mainly involved in the mitochondrial OxPhos pathway in PC. This finding suggests that *SLC25A11* may enhance the rate of OxPhos and promote ATP synthesis in PC cells by participating in the transfer of NADH-reducing equivalents. Furthermore, its expression was found to be negatively correlated with focal adhesion pathway and cell-substrate adhesion. Buffet et al. observed that deletion of *SLC25A11* in a mouse model of metastatic paraganglioma mediated the acquisition of pseudohypoxic features, hypermethylated phenotype, and metastatic properties. They further demonstrated that *SLC25A11* could serve as a novel tumor suppressor gene [29]. Moreover, the expression of ScRNA in the endothelial cells and fibroblasts of PC tissues was found to be considerably different from that in normal tissues. This finding implies that *SLC25A11* may potentially inhibit the transformation of tumors to invasive and metastatic phenotypes in PC.

Additionally, the results of immune infiltration analysis showed that the expression of *SLC25A11* was positively correlated with the recruitment of immune cells, such as CD8 + T, CD4 + T, and DC, which alludes that *SLC25A11* may also play a role in anti-tumor immunity. The carcinogenic effect of *SLC25A11* has been confirmed in lung adenocarcinoma, melanoma, and hepatocellular carcinoma [30, 31]. Our study observed that the OS of patients with a high expression of *SLC25A11* was longer in PC. Similarly, Pan [30] also showed that patients with hepatocellular carcinoma with a high expression of *SLC25A11* had a better prognosis. Therefore, combined with the literature findings, *SLC25A11* can be used as a favorable prognostic marker for PC.

The main physiological role of *SLC25A29* is to transport arginine and lysine to the mitochondria for the synthesis of mitochondrial proteins and the degradation of amino acids. Zhang found that the gene was significantly up-regulated in various cancer cells, especially in cancers with enhanced glycolysis [32]. The knockdown of *SLC25A29* in cancer cells led to a reduction in mitochondrial-derived NO, which resulted in enhanced mitochondrial respiration and reduced glycolysis, thereby reversing the metabolic process. Our study too showed that *SLC25A29* was significantly up-regulated in PC. In addition, enrichment analysis showed that the co-expressed genes in PC were involved in the biological processes of mitochondrial respiratory chain complex assembly, protein extension, and oxidative phosphorylation. PC is mainly characterized by aerobic glycolysis [33], which suggests that *SLC25A29* may cause metabolic remodeling in PC cells by participating in the transport of substrates required for NO synthesis. The results of the co-expression analysis indicated that *SLC25A29* may play a role in suppressing the activation of various immune cells. Analysis of immune infiltration further revealed that the gene may prevent the immune cells from being recruited to confer pro-tumor immunity.

Recent studies have shown that *SLC25A44* is associated with cerebrovascular diseases [34, 35]. The role of this gene in cancer has not been entirely investigated. This study showed that *SLC25A44* is significantly overexpressed in PC and has prognostic value. The results of co-expression analysis explained its role in PC. Genes negatively correlated with *SLC25A44* were found to be mainly involved in adhesion binding, PC, cell cycle, HIF-1, p53, and Hippo pathway in PC. As a member of *SLC25A*, *SLC25A44* has only recently been proven to transport cytoplasmic branched-chain amino acids (BCAAs) to the mitochondrial matrix and be chiefly involved in the catabolism of mitochondrial BCAAs [36, 37]. According to previous literature, in the early stage of pancreatic ductal adenocarcinoma, tissue protein decomposition increases, and systemic circulating BCAAs increase [38]. In a metabolic report on early pancreatic ductal adenocarcinoma, the researchers stated that leucine, a BCAA in acinar cells, is the main source of acetyl CoA [39]. *SLC25A44* facilitates the transport of cytoplasmic BCAAs to the mitochondrial matrix and eventually decomposes them into acetyl-CoA and succinyl-CoA, which are transferred to the TCA cycle. However, the oxidative utilization of BCAAs occurs in a small proportion of PCs, and other shunt pathways exist for the transport of catabolic derivatives of BCAAs to the mitochondria [40]. More detailed metabolic flux analysis is needed to explain it.

Drug sensitivity analysis of SLC25A members showed that the drug sensitivity to SNS-314 increased with the up-regulation of *SLC25A11*. SNS-314 is an aurora enzyme inhibitor that can inhibit tumor growth and enhance chemotherapeutic sensitivity by mediating aurora enzyme activity [41]. A study has reported that AURKA may be a downstream target of the MEK/ERK pathway in PC [42]. This finding suggests that *SLC25A11* may be involved in the MEK/ERK pathway. Cisplatin is one of the standard chemotherapeutic regimens for PC [43]. Our study too found that sensitivity to this drug was up-regulated with the increase in *SLC25A29* expression. SLC25A29 may be involved in the movement of platinum drugs on the cell membrane. In addition, OSI-027 is a selective inhibitor of motor targets [44]. In a xenograft mouse model of PDAC, OSI-027 significantly blocked the G0/G1 phase of the cell cycle and inhibited tumor cell proliferation; furthermore, OSI-027 synergistically enhanced gemcitabine cytotoxicity in vitro and in vivo [45]. Interestingly, in our study, *SLC25A44* was found to enhance the sensitivity to OSI-027. This discovery may form the basis for new clinical combination therapies for PC.

Our study has certain limitations. This study was based on a comprehensive bioinformatics analysis of multiple public databases, which has provided only preliminary insight into the key role of SLC25A members in the progression of PC. However, more research is required to understand how it affects the OxPhos of pancreatic cancer cells and the interactions between OxPhos and the immune microenvironment. Secondly, we focused on the roles of *SLC2511, SLC25A29,* and *SLC25A44* in PC. However, considering the presence of tumor heterogeneity, their potential mechanisms in other cancers must be further analyzed.

## Conclusion

In summary, we first explored the important role of SLC25A members in PC using multiple public databases and bioinformatics techniques. In this study, SLC25A members were observed to be closely associated with the energy metabolism of PC cells, and a prognostic risk model of SLC25A members was established. In addition, *SLC25A11, SLC25A29,* and *SLC25A44* were found to be significantly expressed in PC. Univariate and multivariate lasso–Cox regression analysis showed that these genes had considerable prognostic value in patients with PC and that they could be used as independent prognostic markers. Our results further suggested that *SLC25A11* may be involved in the transfer of mitochondrial NADH reduction equivalents in PC cells and inhibit the phenotypic transformation of PC to invasion and metastasis. Moreover, *SLC25A29* was noted to participate in the metabolic remodeling of PC cells by promoting the synthesis of NO so that the cells get adapted to the harsh environment and survive. Tumor immune analysis revealed that *SLC25A11* and *SLC25A29* are involved in opposing tumor immunity together. Although the mechanism of *SLC25A44* in cancer is unclear, our study found that its expression in PC is negatively correlated with various cancer pathways. Also, *SLC25A44* may potentially act as a BCAA transporter and participate in the energy metabolism of early-stage PC cells. Finally, we predicted that *SCX, NKX2-1*, and TFs are most likely to regulate the mRNA transcription of *SLC25A11, SLC25A29,* and *SLC25A44*. These findings provide novel insights into the progression of PC and its treatment.

## Materials and methods

### Sample collection

We collected RNA-Seq (FPKM) and somatic mutation data on PC samples (TCGA-PAAD) from the TCGA database (https://portal.gdc.cancer.gov/). For pairing with TCGA samples, we downloaded the normal pancreas sample sequencing data from the Genotype-Tissue Expression (GTEx) project (https://xenabrowser.net/hub/) and used Toil Pipeline downloaded from UCSC Xena for homogenization and unification. Furthermore, we collected PC samples (ICGC-PAAD) from the ICGC database (https://daco.icgc.org/) as the validation set. Finally, the RNA-Seq data were uniformly normalized [log2(X + 1)]. The samples with incomplete clinical data were censored.

### DEGs analysis of SLC25A members, co-expression network analysis, and description of clinical features

We used the R software package limma [46] to analyze the gene expression differences between the TCGA-PAAD PC tumor group and the normal tissue group using Log2 [Fold Change (FC)] > 2; P < 0.05; FDR < 0.05 as the standard to identify DEGs of SLC25A members.

Interactions between the protein levels of DEGs of SLC25A members were assessed using STRING (http://string-db.org) [47]. DEGs among different tumor tissue stages were detected by *ANOVA* or two-sided *Wilcoxon* rank-sum test. Finally, according to the median expression level of DEGs, the SLC25A members were assigned to 2 groups: high and low—and their prognostic value in TCGA-PAAD was analyzed by Kaplan–Meier analysis.

### Construction of a new classification of PC and a prognostic risk model by DEGs of SLC25A members

First, we used the R package NMF [48] to undertake a hierarchical clustering analysis based on the DEGs of SLC25A members to identify new classes in PC. Subsequently, PCA of the new classifications was performed, and its prognostic role in PC was analyzed by Kaplan–Meier. Furthermore, we constructed a prognostic risk model for DEGs. Specifically, DEGs of SLC25A members with prognostic values were analyzed through univariate analysis. Integration of the survival time, survival status, and gene expression data was followed by regression analysis using the Lasso-Cox method. In addition, we set up tenfold cross-validation to obtain the optimal model. Finally, we used the Cox multivariate stepwise regression method to filter the final model.

### Correlation analysis of the immune score and metabolic pathways in DEGs prognostic risk model

Based on Bindea's research, we collected 29 gene sets that marked different tumor-infiltrating immune cell types [49]. The single-sample gene set enrichment analysis (ssGSEA) algorithm in the R GSVA package was performed to quantify the level of immune cell infiltration in each PC sample [50]. Subsequently, we obtained gene sets for OxPhos, TCA cycle, and alanine_aspartate_glutamate metabolism from the Kyoto Encyclopedia of Genes and Genomes (KEGG) pathway of GSEA [51]. ssGSEA was used to quantify the activity of each metabolic pathway by scoring and assessing the differences between the high- and low-risk groups.

### Construction and validation of clinical prediction models

To validate the clinical efficacy of the prognostic risk model for DEGs, we evaluated the risk scores in the TCGA-PAAD cohort through univariate and multivariate Cox regression analyses, and nomograms were constructed to assess the prognostic significance of these features. In addition, we built a dynamic nomogram based on the R package Dynnom, which forms an interactive interface for clinical applications, and deployed it on web pages [52]. The ROC analysis was performed using the R package pROC to obtain the AUC of the clinical prediction model. Finally, the survival significance of the model was analyzed by Kaplan–Meier [53].

### Drug susceptibility investigation

We used the CellMiner database (https://discover.nci.nih.gov/cell-miner/home.do) to investigate the expression levels of gene transcriptomes in NCI-60 cell lines and their sensitivity to antitumor drugs [54].

### Co-expression analyses of SLC25A11, SLC25A29, and SLC25A44

We analyzed the DEGs associated with the key SLC25A members transcription in PC based on the LinkedOmics (http://www.linkedomics.org/login.php) functional module. Then, we used Gene Set Variation Analysis (GSVA) to analyze the biological processes and signaling pathways, where the DEGs were concentrated.

### Tumor immune cell infiltration and scRNA analysis of SLC25A11, SLC25A29, and SLC25A44

We also analyzed the differences in the expression levels of SLC25A11, SLC25A29, SLC25A44, and the Spearman correlation of different immune-cell infiltration levels in PC using the TIMER (http://cistrome.shinyapps.io/timer/) database (purity-corrected) [55]. The Tumor Immunity Single-Cell Database (TISCH) (http://tisch.comp-genomics.org/) is a comprehensive scRNA dataset that enables interactive single-cell transcriptome visualization of the tumor microenvironment [56]. In the Datasets module, we visualized the expression levels of SLC25A members at the single-cell level in the PAAD_CRA001160 dataset. Furthermore, differences in the SLC25A members expression in immune cells between the tumor and normal groups were investigated.

### Polygenic TFs prediction

TFs prediction was performed by ChIP-X enrichment analysis 3 (ChEA3) (https://maayanlab.cloud/chea3/) [57]. The ChEA3 database, which contains genomic libraries generated from a variety of sources, is a TFs-enrichment analysis tool that ranks TFs associated with user-submitted genomes.

### Statistical analyses

Data were analyzed using R v 4.0.3. *ANOVA* was used to estimate the statistical significance of differences between the different tumor tissue stages for DEGs that conformed to a normal distribution or a two-sided *Wilcoxon* rank-sum test for non-normal distribution. The Chi-square test or Fisher's exact test was performed to investigate the statistical significance of differences between the categorical variables. Spearman's correlation test was employed to assess two gene correlations as well as gene and drug correlations. One-way and multi-way Cox regression analyses were performed to determine independent prognostic factors. The survival outcomes were assessed using Kaplan–Meier curves, with a two-sided P < 0.05 considered as the threshold of significance.

## Supplementary Information

**Additional file 1: Table. S1.** Mitochondrial solute transporter family 25 differentially expressed genes in pancreatic cancer. **Table. S2.** 10 SLC25A genes by Lasso-cox. **Table. S3.** The top 10 most relevant transcription factors for SLC25A11, SLC25A29 and SLC25A44.

**Additional file 2: Fig. S1.** Exploration of the mRNA and protein level relationships as well as the prognostic value of differentially expressed SLC25A members in PC. (A) Using the string platform, DEGs of SLC25A members were examined for interactions at the protein level. Nodes denote proteins and edges denote specific functional associations between the predicted nodes. (B) The co-expression relationship of DEGs of SLC25A members at the mRNA level was assessed using Spearman’s rank correlation (negative correlation: blue; positive correlation: red), (-P＞0.05, *P＜0.05, **P＜0.01, ***P＜0.001, ****P＜0.0001). (C) According to Kaplan–Meier analysis, seven SLC25 high expressions had a better prognosis, while three SLC25A members low expressions had a better prognosis.

**Additional file 3: Fig. S2.** A new classification based on non-negative matrix decomposition of DEGs and Lasso regression analysis. (A) Non-negative matrix factorization (NMF) clusters were employed to screen for molecular classification, and the results of the ranking survey revealed the optimal grouping of the two subgroups. (B) The heatmap of the consensus matrix of two subgroups. (C) Lasso analysis was used to evaluate 10 candidates’ genes with minimal Lambda.

**Additional file 4: Fig. S3.** Comprehensive analysis of SLC25A11, SLC25A29, and SLC25A44. (A) Co-expressed genes with SLC25A11, SLC25A29, and SLC25A44, respectively, in PC (LinkedOmics). (B) Heatmap displaying the top 50 positively associated genes and the top 50 negatively associated genes co-transcribed with SLC25A11 in PC. (C) SLC25A29, (D) SLC25A44.

## Data Availability

The datasets generated analysed during the current study are available in the ICGC, GTEx and TCGA repository.
