# Supplementary figures and images for: An extensive bioinformatics study on the role of mitochondrial solute carrier family 25 in PC and its mechanism behind affecting immune infiltration and tumor energy metabolism
Source: J Transl Med. 2022 Dec 13;20:592. doi: 10.1186/s12967-022-03756-2 (PMC9746138; doi:10.1186/s12967-022-03756-2)

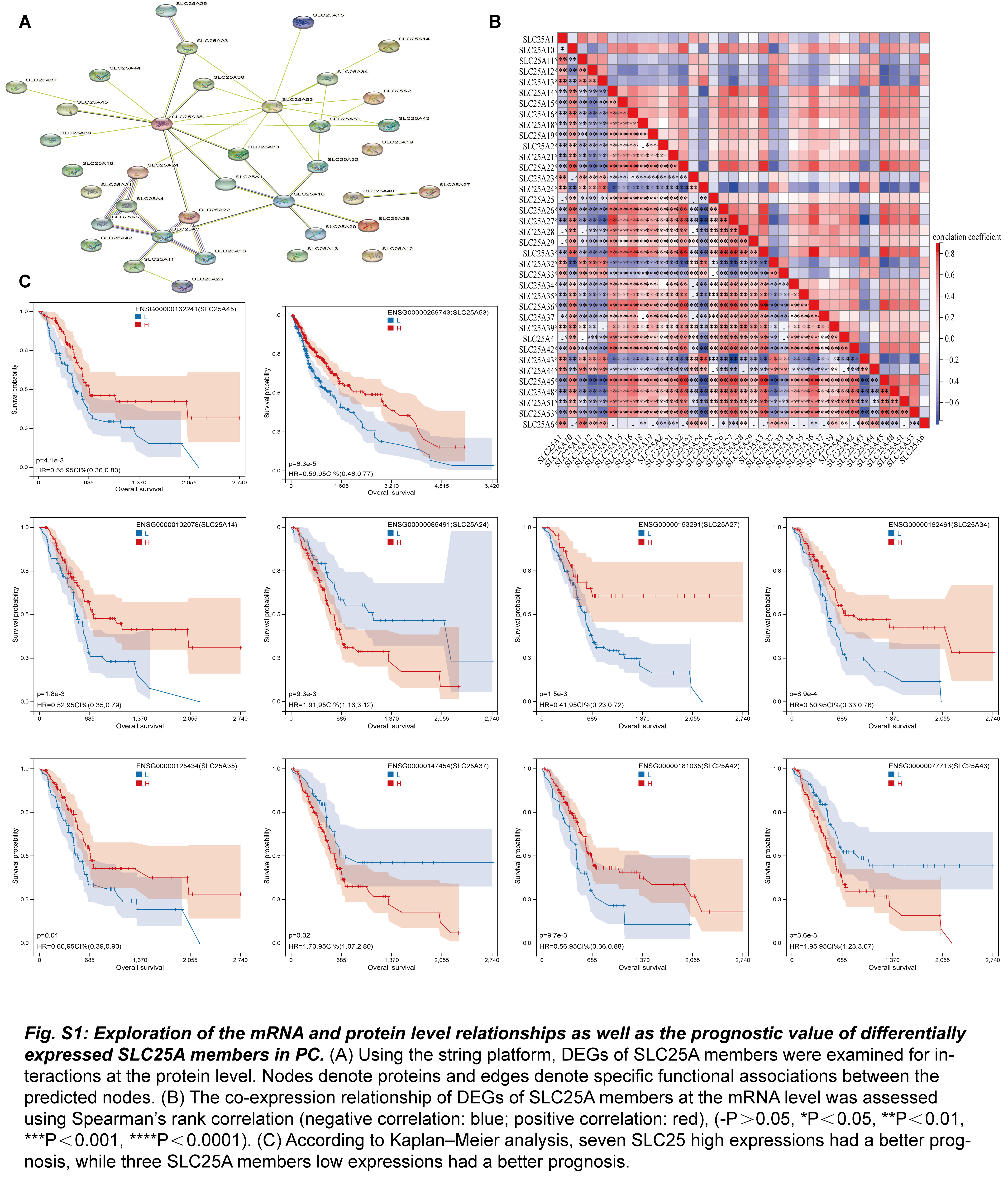

Supplement: Supplementary file 2 — Additional file 2: Fig. S1. Exploration of the mRNA and protein level relationships as well as the prognostic value of differentially expressed SLC25A members in PC. (A) Using the string platform, DEGs of SLC25A members were examined for interactions at the protein level. Nodes denote proteins and edges denote specific functional associations between the predicted nodes. (B) The co-expression relationship of DEGs of SLC25A members at the mRNA level was assessed using Spearman’s rank correlation (negative correlation: blue; positive correlation: red), (-P＞0.05, *P＜0.05, **P＜0.01, ***P＜0.001, ****P＜0.0001). (C) According to Kaplan–Meier analysis, seven SLC25 high expressions had a better prognosis, while three SLC25A members low expressions had a better prognosis. [file 12967_2022_3756_MOESM2_ESM.tif]

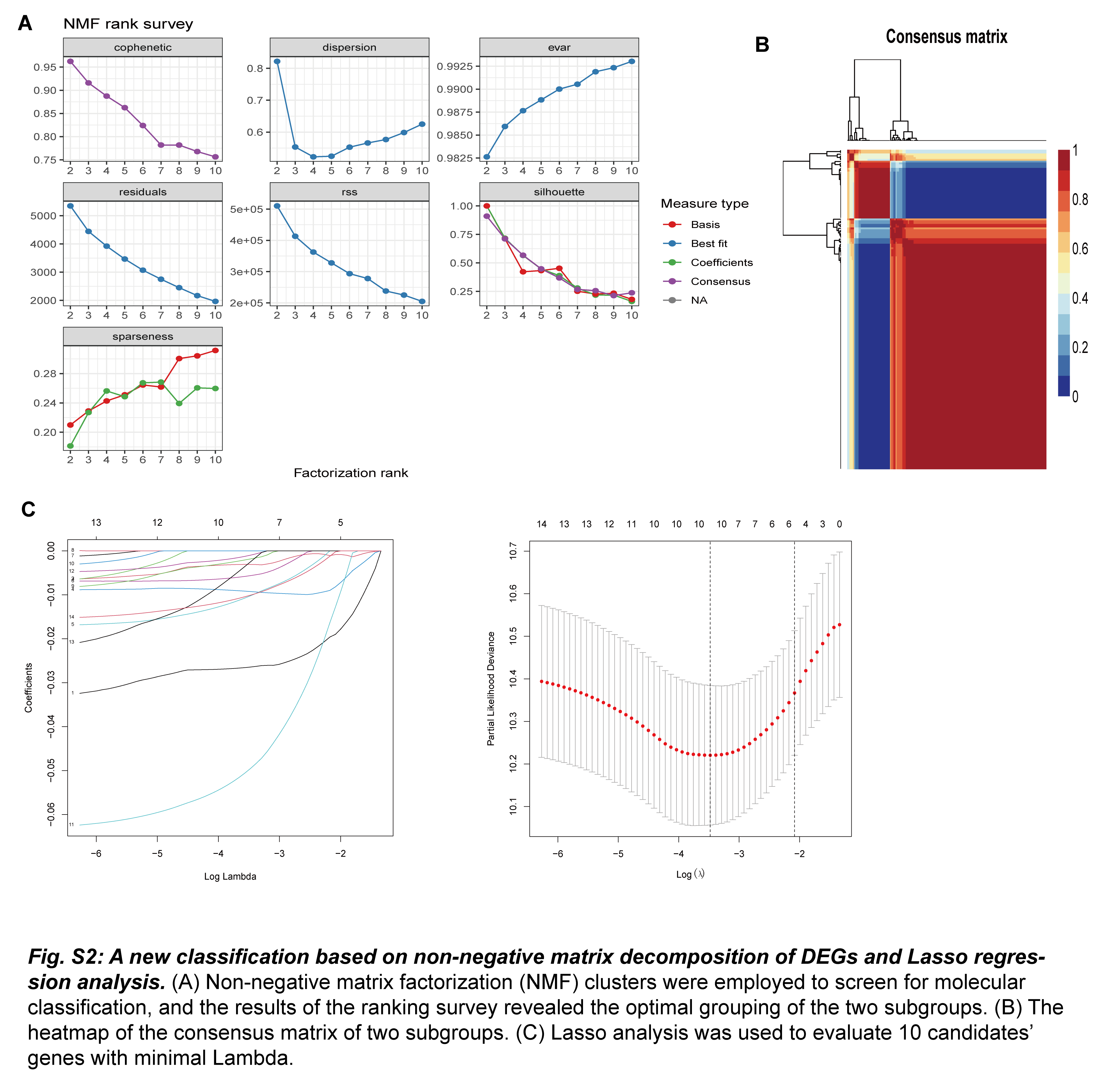

Supplement: Supplementary file 3 — Additional file 3: Fig. S2. A new classification based on non-negative matrix decomposition of DEGs and Lasso regression analysis. (A) Non-negative matrix factorization (NMF) clusters were employed to screen for molecular classification, and the results of the ranking survey revealed the optimal grouping of the two subgroups. (B) The heatmap of the consensus matrix of two subgroups. (C) Lasso analysis was used to evaluate 10 candidates’ genes with minimal Lambda. [file 12967_2022_3756_MOESM3_ESM.tif]

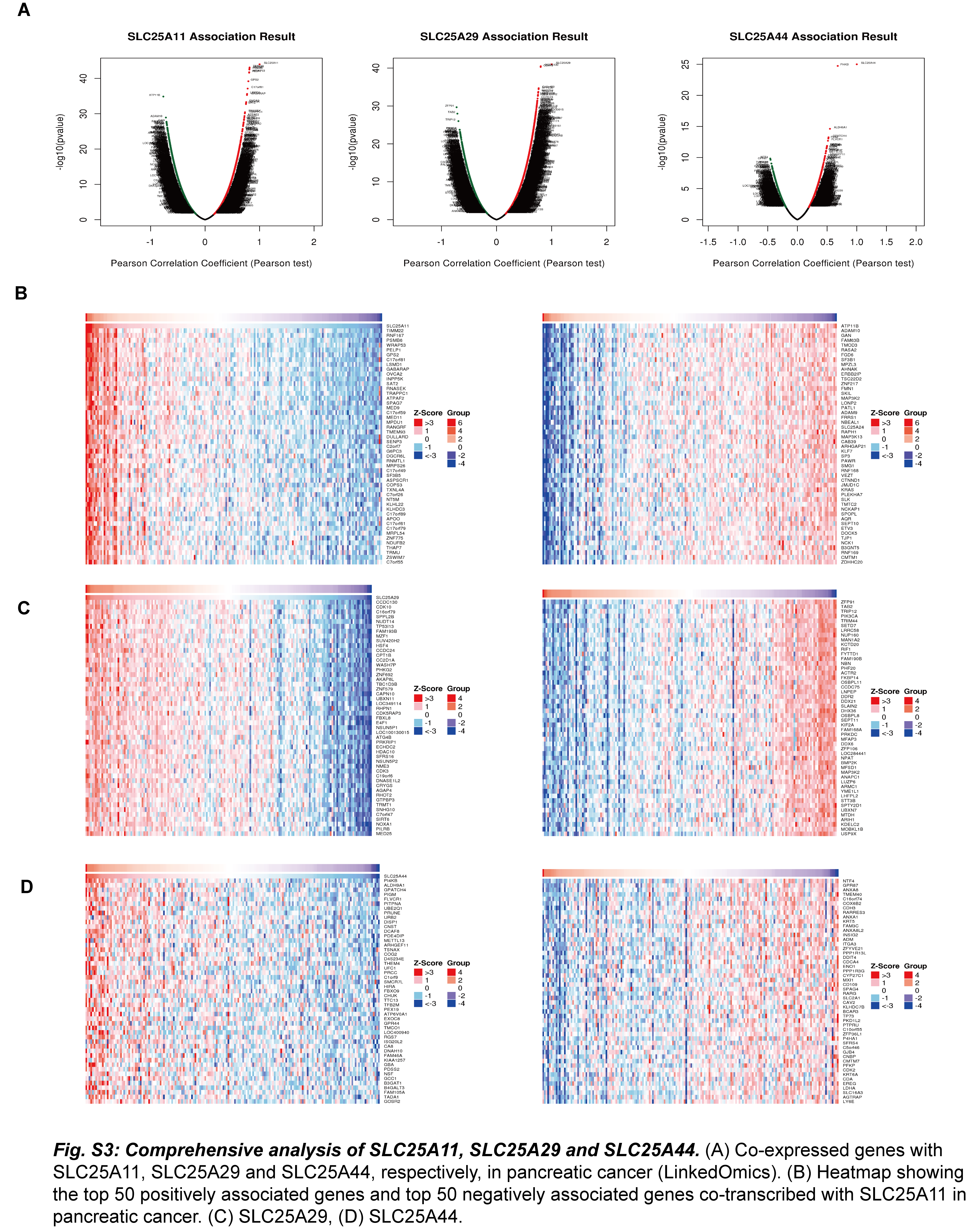

Supplement: Supplementary file 4 — Additional file 4: Fig. S3. Comprehensive analysis of SLC25A11, SLC25A29, and SLC25A44. (A) Co-expressed genes with SLC25A11, SLC25A29, and SLC25A44, respectively, in PC (LinkedOmics). (B) Heatmap displaying the top 50 positively associated genes and the top 50 negatively associated genes co-transcribed with SLC25A11 in PC. (C) SLC25A29, (D) SLC25A44. [file 12967_2022_3756_MOESM4_ESM.tif]
